# Influence of Curved Video Laryngoscope Blade Sizes and Patient Heights on Video Laryngoscopic Views: A Randomized Controlled Trial

**DOI:** 10.3390/jpm14020209

**Published:** 2024-02-15

**Authors:** Jong-Ho Kim, Bo-Reum Cheon, Hyesook Kim, Sung-Mi Hwang, Jae-Jun Lee, Young-Suk Kwon

**Affiliations:** 1 Department of Anesthesiology and Pain Medicine, Chuncheon Sacred Heart Hospital, College of Medicine, Hallym University, Chuncheon 24253, Republic of Korea; poik99@hallym.or.kr (J.-H.K.); qhxoddl15@hallym.or.kr (B.-R.C.); h70sm@hallym.or.kr (S.-M.H.); 2 Institute of New Frontier Research, College of Medicine, Hallym University, Chuncheon 24253, Republic of Korea; hskim87@hallym.ac.kr

**Keywords:** laryngoscope, body height, video-assisted techniques, randomized controlled trial, endotracheal intubation

## Abstract

This study aimed to compare the video laryngoscope views facilitated by curved blades 3 and 4 with an exploration of the relationship between these views and patient height. Conducted as a randomized controlled trial, this study enrolled adults scheduled for surgery under general anesthesia. Intubation procedures were recorded, and the percentage of glottic opening was measured before tube insertion. Multivariate analysis validated the impact of various factors, including blade size and patient height, on the percentage of glottic opening scores. A total of 192 patients were included. The median percentage of glottic opening scores for curved blades 3 and 4 were 100 and 83, respectively (*p* < 0.001). The unstandardized coefficient indicated a significant negative impact of blade 4 on the percentage of glottic opening scores (−13, *p* < 0.001). In the locally estimated scatterplot smoothing analysis, blade 3 exhibited a steady rise in glottic opening scores with increasing height, whereas blade 4 showed a peak followed by a decline around 185 cm. The unstandardized coefficient of height showed no significant association (0, *p* = 0.819). The study observed superior laryngoscopic views with blade 3 compared to blade 4. However, no significant association was found between laryngoscopic views and patient height.

## 1. Introduction

A video laryngoscope is an indirect laryngoscopic tool capable of visualizing airway structures, including the vocal cords, without a direct line of sight. A video laryngoscope helps practitioners see things that cannot be seen within sight. Video laryngoscopes enable other practitioners to see what video laryngoscope operators see, which provides opportunities for cooperation and education [1,2]. Importantly, video laryngoscopy is increasingly widely used because video laryngoscopes can improve the success of intubation [3,4,5]. 

Some physicians argue that blade size 3 is usually used, whereas blade size 4 is suitable only for patients who are overweight or have a very long thyromental distance in conventional laryngoscopy [6]. It has also been reported that blade 4 can improve the view during direct laryngoscopy in patients with a large lower jaw or deep pharynx [7]. In contrast, some clinicians recommend using blade 4 first in all adult patients, considering that the vertical flange height is similar between blade sizes 3 and 4 in conventional laryngoscopy [8]. 

The video laryngoscopes can be used directly and indirectly and are produced by several manufacturers [9,10,11]. The Macintosh blade is one of the most commonly used curved blades in conventional laryngoscopy and is easy to manipulate for an experienced anesthesiologist [12,13]. In curved blades like Macintosh blades, practitioners generally prefer blade sizes 3 and 4 in conventional laryngoscopy [14]. In recent studies involving critically ill patients, it has been reported that blade 4 was used more than blade 3 in conventional Macintosh laryngoscopy [15,16]. However, what size fits which patients is not well known in curved video laryngoscope blades. Furthermore, studies on the effects of the size of curved video laryngoscope blades are limited. Even in the manufacturer’s instructions, the subjects of blade sizes 3 and 4 were described as adults who were ambiguously distinguished (e.g., medium or large adults) [17,18,19]. Therefore, the criteria for selecting the appropriate curved video laryngoscope blade size are unclear. In the previous study, tracheal intubation was performed only by an experienced anesthesiologist [13], whereas in the remaining three studies, the degree of skill or expertise of the practitioners attempting intubation was not known [14,15,16]. Consequently, there is a lack of data on size selection by experienced anesthesiologists in video laryngoscopy. Therefore, investigating factors influencing blade selection becomes even more crucial. Because of this issue, identifying factors that can help select the appropriate size of the curved video laryngoscope blade is essential.

The study aimed to assess how curved video laryngoscope blade sizes 3 and 4 influence the video laryngoscope view. Additionally, it aimed to explore the distribution of video laryngoscope views concerning patient height. Closing this knowledge gap is essential to better define the criteria for selecting the most suitable curved video laryngoscope blade size, ultimately improving airway management in clinical settings.

## 2. Materials and Methods

### 2.1. Study Setting

This randomized controlled trial was approved by our Institutional Review Board (No. 2021-10-011) and was registered before patient enrollment at https://cris.nih.go.kr (accessed on 13 December 2023) (KCT0006820). Written informed consent was obtained from all participants. This study was conducted from November 2021 to March 2023.

### 2.2. Participants, Study Design, and Randomization

In this study, adult patients, aged ≥ 18 years, who were scheduled for elective surgery with general anesthesia, were screened. The exclusion criteria were as follows: patients who have undergone or have plans to undergo airway-related surgery; intubated patients; those without informed consent to the study; those with cervical instability; those requiring rapid sequence intubation; and those who were contraindicated to undergo video laryngoscopy.

According to instructions from specific manufacturers, including VERHON’s GlideScope (Verathon Medical, Burnaby, BC, Canada) and BESDATA’s video laryngoscope (Besdata, Shenzhen, China) [19,20], blade 4 was described as being used in large adults. According to our medical records, the height of the shortest patient for whom only blade 4 was used was 169 cm. Recognizing the ambiguity in the manufacturers’ definition of large adults and aiming to minimize the need for video laryngoscope reinsertions and prevent potential injury due to excessively large blade sizes, we established a practical criterion. To maintain statistical convenience, we decided to set a threshold at 170 cm, including patients with heights greater than or equal to 170 cm, for participation in this randomized controlled trial. In other words, we conducted our study with a focus on patients whose height exceeded 170 cm, aligning with the practical considerations derived from the minimum height (169 cm) observed in our medical records, where blade 4 was exclusively used. This approach was taken due to the challenge of precisely defining ‘large adults’ according to manufacturers’ guidelines and our commitment to ensuring patient safety during the intubation process. This study was a block-randomized, parallel-group trial with two equal-sized groups. The participants were randomly assigned to one of the two groups: the curved video laryngoscope blade size 3 group or the curved video laryngoscope blade size 4 group. A randomization chart was developed using a computer-generated randomization system with a block size of four. The allocation ratio was set at 1:1. Randomization was performed by the anesthesiologists, who prepared individual opaque, sealed envelopes for all participants, containing blades with computer-generated sizes for all allocations. On the day of surgery, the opaque, sealed envelopes were sent to the operating room by an anesthesia nurse who was not involved in the patients’ perioperative care. The assigned anesthesiologist opened the envelopes and performed intubation.

As the two blade sizes, blades 3 and 4, differed, the anesthesiologist who performed intubation was aware of the sizes of the blades used. A blind statistician did not directly participate in the allocation of patients or collecting data. In principle, the patients and the surgery team were blinded to the group allocation.

### 2.3. Primary and Secondary Outcomes

The primary outcome was the percentage of glottic opening score, which was assessed using the video laryngoscope view. The range of measurement was from the interarytenoid notch to the anterior commissure. The percentage of the glottic opening score represents the percentage of the glottis that is visible during laryngoscopy. A percentage of the glottic opening score of 100 indicates a visualization of the entire glottis, from the anterior commissure of the vocal cords to the interarytenoid notch. If the glottic opening is not visualized, the percentage of the glottic opening score would be 0 [21,22].

During video laryngoscope manipulation, a video clip was recorded. The video laryngoscope view was captured just before the endotracheal tube passed the vocal cords, and the percentage of glottic opening score was measured from the captured image. In patients with a percentage of glottic opening scores of 0, the percentage of glottic opening score when lifting up the epiglottis or applying the Backwards, Upwards, Rightwards, and Pressure techniques was also measured and recorded. Our investigation focused on comparing the percentage of glottic opening scores between blades 3 and 4 and assessing the distribution of these scores according to patient height within each blade size. The secondary outcomes included the number of intubation attempts and the intubation success rate.

### 2.4. Preoperative Measurement

To control the other factors associated with a difficult airway, we measured the modified Mallampati class [23], thyromental height [24], thyromental distance [5,25], sternomental distance [26], distance with the mouth open [27], and neck circumference [27,28]. These factors, except for thyromental height, were measured with the patients placed in the sitting position. The thyromental height was measured with the patient placed in the supine position. All measurements were performed by one research nurse. The research nurse was blinded to the group allocation.

### 2.5. Intubation

**Figure 1:**
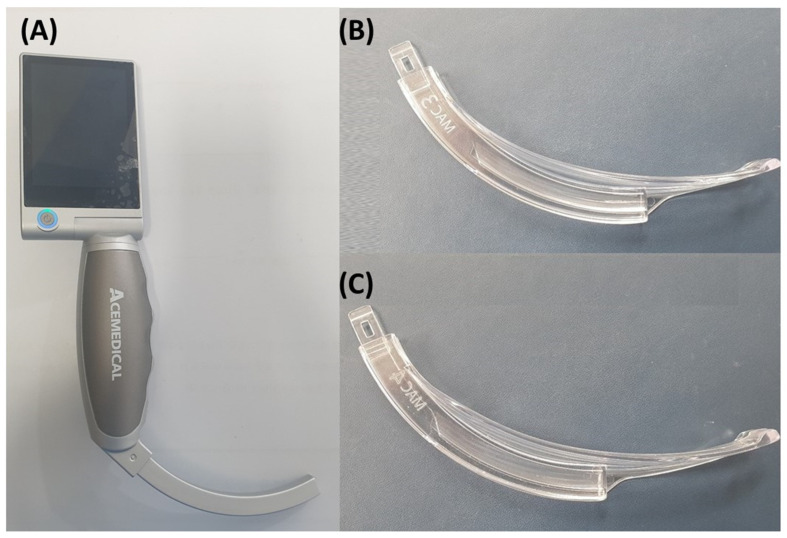
Video laryngoscopy and blade. (**A**) Video laryngoscope; (**B**) curved video laryngoscope blade 3; and (**C**) curved video laryngoscope blade 4.

Intubation procedures were exclusively performed by an airway expert—an experienced anesthesiologist with over 8 years of proficiency in airway management and anesthesia—utilizing Acemedical’s AceScope video laryngoscope (Acemedical, Seoul, Republic of Korea) (Figure 1). The specifications for the AceScope video laryngoscope indicate a resolution of 640 × 480 pixels at a maximum frame rate of 30 frames per second, an angle of view/depth of field of approximately 71.9 degrees diagonally, with an allowable range of variation of approximately ±15%, and a light source of camera LED providing over 150 lux illumination. Additional technical details such as storage type, display specifications, power source, and operating/charging time can be found in Supplementary Table S1. The anesthesiologist selected either a curved video laryngoscope blade size 3 or 4 based on group allocation. General anesthesia was induced using propofol and rocuronium by an assigned anesthesiologist. Endotracheal intubation took place upon reaching a train-of-four value of 0. 

In our study, the depth of insertion of the blade was rigorously controlled to ensure consistency across all trials. Specifically, our standardized approach aimed to insert the tip of the blade precisely into the patient’s vallecula, regardless of the specific blade size used. 

**Figure 2:**
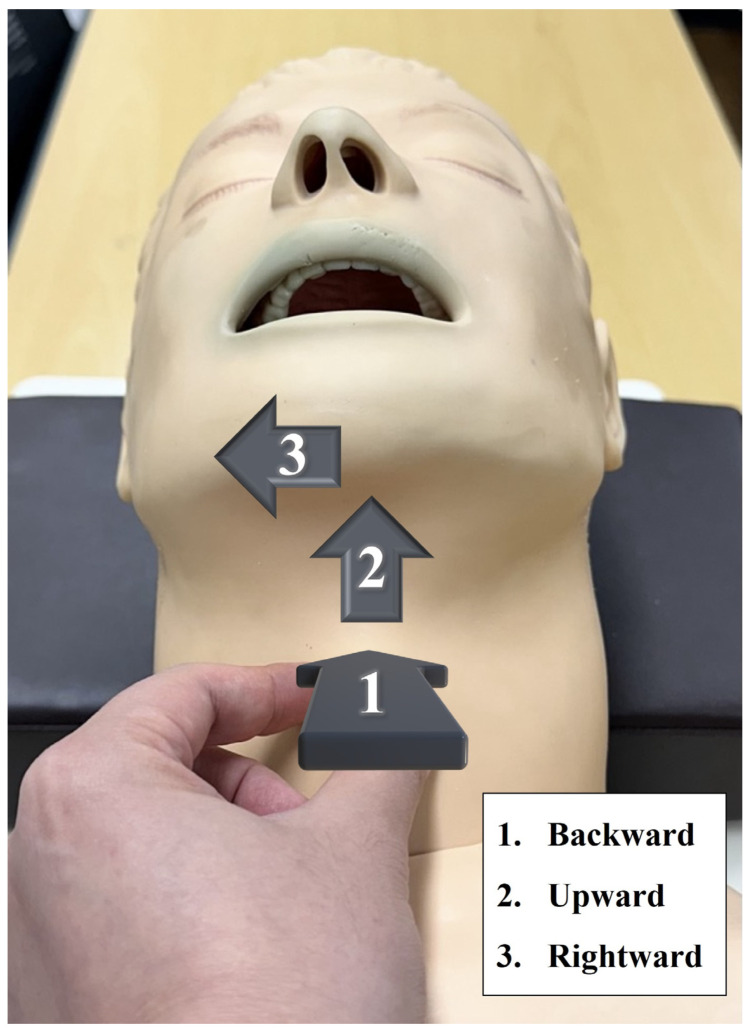
BURP maneuver. BURP: Backwards, Upwards, Rightwards, and Pressure.

On the intubation protocol, when the percentage of glottic opening score was 0 and tracheal intubation did not seem easy, a method to lift up the epiglottis would be used without withdrawing the video laryngoscope. After that, the Backwards, Upwards, Rightwards, and Pressure techniques would be used without withdrawing the video laryngoscope (Figure 2). If failed intubation were to occur after performing the aforementioned methods, other appropriate approaches were considered. It was observed that the count of intubation attempts increased when the video laryngoscope was temporarily removed and retried, or when an alternative method was attempted.

### 2.6. Statistical Analysis

Previous studies have assumed a 25% difference in the percentage of glottic opening scores [29], but there is a lack of clear consensus on whether this difference is clinically meaningful in all situations. Therefore, we calculated the sample size based on the percentage of glottic opening scores according to blade size in a pilot study using G*Power (version 3.1.9.6; Kiel University, Kiel, Schleswig-Holstein, Germany). Input parameters were as follows: effect size (Cohen’s d) = 0.607, α = 0.05, power = 0.95, and allocation ratio = 1. After calculations, we aimed for each group to comprise 75 patients, setting a total of 200 individuals to account for an anticipated dropout rate of 25%. Continuous variables were presented as means with standard deviations or as medians with interquartile ranges. Statistical analysis employed the t-test or Mann–Whitney test for continuous variables, while categorical variables were expressed as counts and percentages and analyzed using the chi-square test or Fisher’s exact test.

The distribution of the percentage of glottic opening score concerning blade size was examined using histograms. Furthermore, we assessed the distribution of the percentage of glottic opening in each blade size according to patient height. Additionally, a locally estimated scatterplot smoothing line was applied to illustrate a smooth curve depicting the relationship between height and the percentage of glottic opening score, aiding in comprehending the association.

The multivariable linear regression model validated the impact of various factors, including blade size and patient height, on the percentage of glottic opening scores. Statistical analyses were performed using the Statistical Package for the Social Sciences (version 26.0; IBM Corp., Armonk, NY, USA), and all tests were two-sided with a predetermined alpha value of 0.05.

## 3. Results

**Figure 3:**
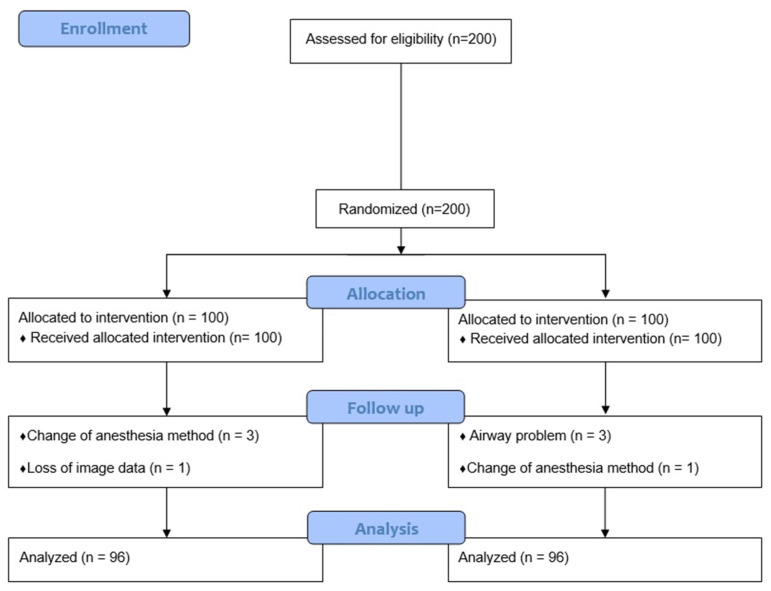
Flow chart.

Two hundred patients were enrolled according to the exclusion criteria in this study. After the follow-up loss of eight patients, the remaining 192 patients were analyzed. The details of the participants are summarized in Figure 3. Each group included 96 patients. No significant differences in the demographic data were observed between the two groups. The details of the patient’s characteristics are summarized in Table 1.

### 3.1. Percentage of Glottic Opening Scores According to Blade Size

**Figure 4:**
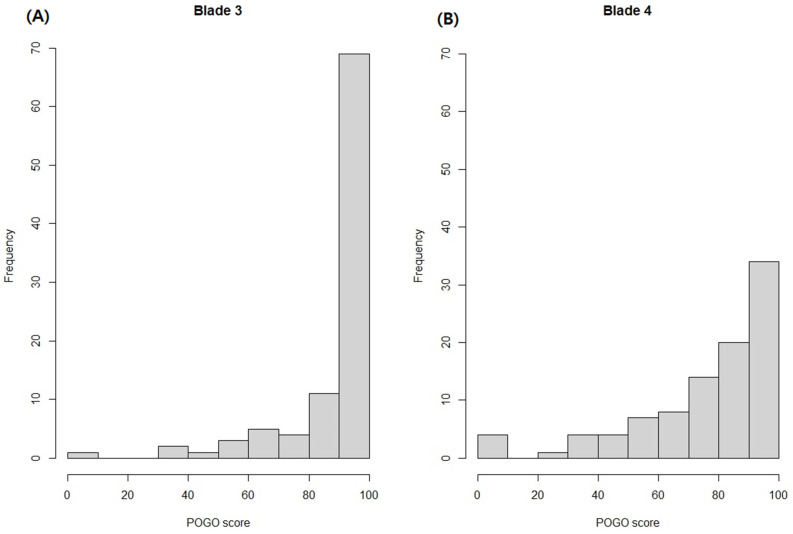
Distribution of percentage of glottic opening scores according to blade size. (**A**) Blade 3 and (**B**) blade 4.

The median percentage of glottic opening scores in the blade 3 and 4 groups were 100 (interquartile range (IQR), 87–100) and 83 (IQR, 63–100), respectively. A statistically significant difference in the median percentage of glottic opening scores was noted between the two groups (*p* < 0.001). Within the blade 3 group, only one patient (1.1%) had a percentage of glottic opening score of 0, which improved to 50 when the epiglottis was lifted. Comparatively, in the blade 4 group, four patients (4.3%) exhibited a percentage of glottic opening score of 0, with corresponding scores of 95, 65, 60, and 26 upon lifting the epiglottis. The percentage of glottic opening scores of 100 was reported in 66 patients (71.7%) and 33 patients (35.9%) in the blade 3 and 4 groups, respectively. Notably, none of the patients required the Backwards, Upwards, Rightwards, or Pressure techniques. Figure 4 depicts the distribution of the percentage of glottic opening scores based on blade size.

### 3.2. Percentage of Glottic Opening Score and Patient Height

**Figure 5:**
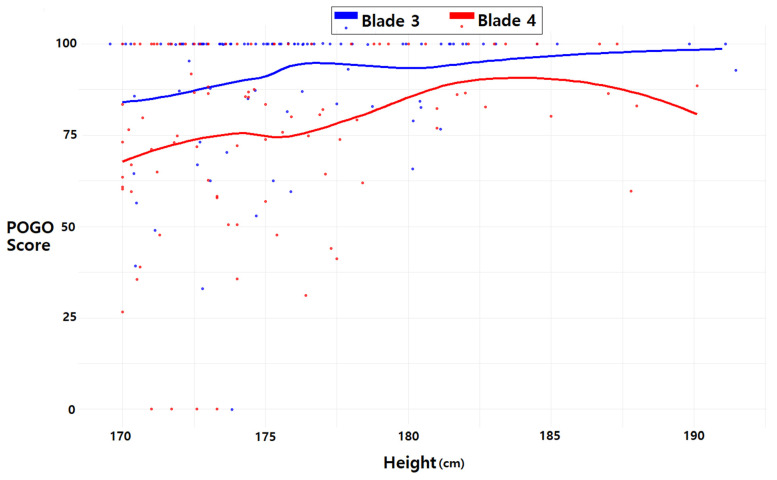
Relationship between patient height and percentage of glottic opening score according to blade size. The dots are scatterplots of patient height and percentage of glottic opening score. The lines are estimated scatterplot smoothing lines. POGO, percentage of glottic opening.

In Figure 5, the distribution of the percentage of glottic opening scores according to patient height is presented for each blade size. For blade 3, the locally estimated scatterplot smoothing lines indicate a gradual rise in the percentage of glottic opening scores corresponding to height. In contrast, for blade 4, there is an initial increase in the percentage of glottic opening scores with height, followed by a decline around 185 cm.

### 3.3. Association of Percentage of Glottic Opening Score with Blade Size and Patient Height by Multiple Linear Regression Analysis

The unstandardized coefficient of blade 4 (reference: blade 3) and patient height for percentage of glottic opening score were −13 (−19–−7) and 0 (−1–1) when all variables were included, respectively. In the context of this study, the unstandardized coefficients in the multiple linear regression analysis indicate the mean effect of each airway variable on the change in POGO score. The details of the unstandardized coefficient, including other variables and after backward elimination, are summarized in Table 2.

### 3.4. Secondary Outcomes

Among the total patients, only one patient using blade 4 had two intubation attempts, and the remaining patients had one intubation attempt (*p* > 0.999). No patients failed endotracheal intubation.

In instances where two intubation attempts were necessary, we opted to switch from blade 4 to blade 3 to prevent the potential airway injury associated with forceful intubation using blade 4. In the first attempt, the patient had a percentage of glottic opening score of 0 and a percentage of glottic opening score of 26 when the epiglottis was lifted up. When blade 3 was used, the percentage of glottic opening score before and after lifting up the epiglottis was 34 and 67, respectively.

## 4. Discussion

In this randomized controlled trial, we investigated and compared the video laryngoscope field of view between blade sizes 3 and 4 in video endoscopy. Our findings revealed that using a curved video laryngoscope, blade 3 offered a notably superior field of view compared to blade 4, as indicated by the percentage of glottic opening score during video laryngoscopy. However, it is important to note that endotracheal intubation was successfully achieved in the first attempt for nearly all patients enrolled in the study. In the majority of cases, the percentage score for glottic opening was above 60, regardless of blade size. Observing blade 3, the percentage score for glottic opening increased proportionally with patient height, whereas for blade 4, the score initially ascended with height and later exhibited a decline around 185 cm. Notably, the patients with a glottic opening score of zero were all below 175 cm in height.

Recent multicenter studies investigating the relationship between Cormack–Lehane grades and conventional Macintosh laryngoscope blade sizes in critically ill or emergency department patients reported higher intubation success rates with blade size 3 compared to blade size 4. However, intriguingly, discrepancies existed between these studies. While one study found no disparity in the Cormack–Lehane grade between blade sizes [15], another reported a difference [16]. In our study, a percentage of glottic opening score of 100 was frequently observed, and a percentage of glottic opening score of 0 was not common for video laryngoscope views with either blade size 3 or 4. The success rate in the first attempt was higher in our study, with no significant difference observed between blade sizes.

These deviations from previous studies might be attributed, in part, to the utilization of a video laryngoscope, which generally yields an improved laryngoscopic view compared to conventional laryngoscopy [30,31,32,33]. Additionally, it is noteworthy that the level of operator experience, both inside and outside the operation room, could have influenced the outcomes. Operators outside the operation room who participated in our study may have had varying levels of experience, potentially contributing to the observed differences from studies conducted within the OR setting.

Attaining an optimal laryngoscopic view holds paramount importance during endotracheal intubation. A suboptimal view can lead to multiple intubation attempts, prolonged intubation time, airway trauma, and even intubation failure [34,35]. This becomes especially critical for non-specialists in airway management due to their limited experience in achieving a better laryngoscopic view, potentially resulting in complications [36,37]. Blade size 4, being longer than blade size 3, may pose challenges in approaching the vallecular region and lifting the epiglottis with a shorter blade. Conversely, a longer blade might inadvertently visualize the proximal esophagus, potentially leading to inadvertent esophageal intubation [15]. Hence, the selection of the appropriate blade size remains crucial. Our study underscores that the video laryngoscope view varies among airway management experts depending on the curved video laryngoscope blade size. Nevertheless, a video laryngoscope consistently provides a sufficient view for endotracheal intubation, with a rare occurrence of a poor view, irrespective of blade size. 

Despite several methods proposed for choosing the appropriate blade size in conventional laryngoscopy, such as thyromental distance, weight, and upper incisor to hyoid bone distance [6,38,39], a definitive method remains elusive. Manufacturer instructions ambiguously categorize Macintosh curved blades 3 and 4 for “medium-sized” and “large” adults, respectively [18]. Height is generally an evaluation criterion for human size, and height-based selection of blade size has been used for customary double-lumen tubes in endotracheal intubation [40]. While height is often a criterion for assessing human size, we acknowledge that the existing literature may not extensively associate patient height directly with the likelihood of success in visualization. Our study was motivated by the common practice of using patient height as an evaluative factor in airway management. In the multivariate analysis of this study, no significant effect of patient height on the percentage of glottic opening scores associated with video laryngoscope blade size was detected. In addition, the clinical significance of these findings may not be substantial due to the generally high percentage of glottic opening scores in our study results. However, in certain scenarios, specific factors may influence blade size selection. Particularly, we observed that glottic opening scores of 0 occurred in patients shorter than 175 cm, and 80% of these occurrences were observed with blade size 4. Despite the limited number of cases, our study findings suggest that the risk of encountering a percentage of glottic opening score of 0 may be increased when using blade size 4 in patients who are shorter in height.

In this study, laryngeal view was assessed using the percentage of glottic opening scores as the primary outcome, but the success of endotracheal intubation on the first attempt was also recorded as an important secondary outcome. However, it is important to note that there may be a potential discrepancy between the laryngeal anatomy visualized using the video laryngoscope, especially the Acescope used in this study, and the successful passage of the endotracheal tube measured by the percentage of glottic opening scores. Although our study emphasized the evaluation of laryngeal view, future research related to video laryngoscopes should equally prioritize the successful insertion of the endotracheal tube, recognizing that a clear laryngeal view may not always guarantee smooth tube insertion. This can ensure a more comprehensive evaluation of the effectiveness of intubation.

In our study, a 100% success rate in tracheal intubation was achieved using a video laryngoscope. While the impact of blade size and patient height on the success of tracheal intubation may not hold significant clinical relevance, variations in laryngoscopic views, particularly instances of poor visualization, could carry several noteworthy clinical implications. Generally, when the laryngoscopic view is poor during endotracheal intubation, it can lead to prolonged intubation times compared to situations where the laryngoscopic view is good [41,42]. A poor laryngoscopic view makes it more challenging for healthcare providers to visualize the vocal cords and navigate the endotracheal tube into the trachea, thus potentially increasing the time required for successful intubation. Several factors contribute to prolonged intubation time with a poor laryngoscopic view:

Difficulty in visualizing anatomy: A poor view makes it harder to properly visualize the airway anatomy, including the vocal cords and surrounding structures, which can lead to delays in tube insertion [43,44].

Need for repeated attempts: Healthcare providers might require multiple attempts to achieve successful intubation when the view is inadequate. Each attempt takes time and can increase the overall intubation duration [45].

Increased risk of complications: Prolonged attempts due to poor visualization can increase the risk of complications associated with intubation, such as tissue trauma, hypoxia, or aspiration [46,47].

Reduction in oxygenation and ventilation: A longer intubation time may reduce effective oxygenation and ventilation, which is crucial in critically ill patients [48].

Efforts to improve laryngoscopic view, such as using proper laryngoscope blades, reduce intubation times and minimize complications associated with challenging intubations. 

Among the patients assigned to the blade 4 group, three were dropped in this study due to airway problems. Blade 4 is longer by approximately a few centimeters and a few millimeters wider than blade 3, depending on the manufacturer [17]. In this study, blade 4 was longer by approximately 1 cm and 3 mm wider than blade 3. When both ends of the blades were placed on the floor, the difference in height from the floor was approximately 5 mm. In this study, blade 4 was too bulky to insert for those who had small mouths and loose teeth. The larger width of the blade may limit the insertion of the blade in patients with limited mouth openings [15]. As a result, patients who cannot use blade 4 due to its bulkiness have no choice but to use blade 3.

To the best of our knowledge, this is the first study to investigate the video laryngoscope view depending on curved video laryngoscope blade size and the effect of patient height on the video laryngoscope view. However, this study had some limitations. First, the samples in this study were limited. The subjects were all Asian (specifically Korean) adults, and almost all were male according to the eligibility criteria of height. The pharynx and larynx are generally smaller in Asians than in Caucasians [49]. Generally, males have larger airways than females, in terms of both diameter and length [50]. The vocal cords, which are part of the upper airway, are longer and thicker in males than in females [51,52]. There may be differences in the results involving other races or groups, including many females. Second, the video laryngoscope characteristics may be different depending on the manufacturer. In particular, the width and length of the blade are various, which subsequently affect the view angle. The viewing angle of the blade can affect the visualization of the airway [48]. Therefore, the generalizability of our findings to other video laryngoscopes may be limited. Third, all intubation procedures were performed by an expert. Therefore, our results are not appropriate to apply to less experienced practitioners of airway management. For less experienced practitioners of airway management, a good laryngoscopic view does not result in the success of intubation. Furthermore, they are more likely to achieve a poor laryngoscopic view than experts [53,54]. Forth, this study was designed as a block-randomized trial, but it was not possible to completely blind the anesthesiologists performing intubation to the blade sizes used in video laryngoscopy. This could have influenced their technique and the results of the study. The researcher assessing the percentage of glottic opening scores was blinded to the group allocation, but it was still possible for them to be biased by their knowledge of the blade sizes. The visibly distinct blade sizes might have affected both the recorded video laryngoscope views and the subsequent percentage of glottic opening score measurements. This means that the results of the study should be interpreted with caution. Fifth, while this study sheds light on the potential relationship between blade size, patient height, and POGO scores, its clinical significance is limited. Larger-scale investigations are warranted to delve deeper into their clinical relevance and establish definitive recommendations.

## 5. Conclusions

Our study suggests that for adults over 170 cm, initiating intubation attempts with a curved video laryngoscope blade size 3 may be an effective starting point for achieving a favorable video laryngoscope view. Our findings indicate that a larger curved video laryngoscope blade size may not always be the most effective choice for optimizing the video laryngoscope view in tall patients. This suggests a strategic approach to selecting the appropriate blade size: starting with a size 3 blade and transitioning to a size 4 blade if necessary to achieve optimal visualization. In addition, further research is needed to validate the generalizability of our conclusions across different video laryngoscopes. Future studies should aim to evaluate and compare different models to comprehensively understand their performance in diverse patient populations and clinical scenarios.

## Figures and Tables

**Table 1:** Demographic data and patients’ characteristics.

	Blade 3 (*n* = 96)	Blade 4 (*n* = 96)	*p* Value
Age, years (mean [SD])	44.1 (16.0)	47.3 (16.7)	0.173
Male (number [%])	92 (95.8)	91 (94.8)	0.733
Patients with Mallampati class > 2, (number [%])	13 (13.5)	13 (13.5)	>0.999
Thyromental height, cm (mean [SD])	5 (1.0)	5.1 (1.0)	0.301
Thyromental distance, cm (mean [SD])	9.6 (1.3)	9.9 (1.3)	0.183
Sternomental distance, cm (mean [SD])	17.1 (2.0)	16.9 (1.8)	0.487
Mouth opening, cm (mean [SD])	5.7 (0.8)	5.7 (0.7)	0.816
Neck circumference, cm (mean [SD])	40.4 (3.2)	40.4 (2.7)	0.951
Height, cm (mean [SD])	175.9 (4.5)	175.5 (5.0)	0.505
Weight, kg (mean [SD])	81.5 (16.4)	80.7 (12.9)	0.710

SD, standard deviation.

**Table 2:** Airway variables and their impact on the percentage of glottic opening score: a multiple linear regression analysis.

Variables	Full Variables Model		Backward Elimination Model	
	Unstandardized Coefficient (95% Confidence Interval)	*p* Value	Unstandardized Coefficient (95% Confidence Interval)	*p* Value
Blade size 4 (reference: blade size 3)	−13 (−19–−7)	<0.001	−13 (−19–−7)	<0.001
Height (cm)	0 (−1–1)	0.819	Eliminated	
Age (year)	0 (−1–−0.28)	<0.001	0 (−1–0)	<0.001
Female	6 (−9–21)	0.433	Eliminated	
Mallampati class	−1 (−4–3)	0.684	Eliminated	
Thyromental height (cm)	0 (−3–4)	0.791	Eliminated	
Thyromental distance (cm)	3 (−0–5)	0.06	3 (0–6)	0.003
Sternomental distance (cm)	1 (−1–3)	0.149	1 (0–3)	0.146
Mouth opening (cm)	2 (−2–6)	0.252	Eliminated	
Neck circumference (cm)	0 (−2–1)	0.625	−1 (−2–0)	0.108
Weight (kg)	0 (0–0)	0.649	Eliminated	

Unstandardized coefficient: the mean effect of each airway variable on the change in percentage of glottic opening score.

## Data Availability

The data presented in this study are available upon request from the corresponding author. The data are not publicly available due to institutional policy.

## Supplementary Materials

The following supporting information can be downloaded at: https://www.mdpi.com/article/10.3390/jpm14020209/s1, Table S1: Technical specification of using Acemedical Acescope.
