# Supplementary material for: Influence of Curved Video Laryngoscope Blade Sizes and Patient Heights on Video Laryngoscopic Views: A Randomized Controlled Trial
Source: J Pers Med. 2024 Feb 15;14(2):209. doi: 10.3390/jpm14020209 (PMC10889943; doi:10.3390/jpm14020209)
Supplement: Supplementary file 1 [file jpm-14-00209-s001.zip › jpm-2876074-supplementary.pdf]

Supplementary table S1. Technical specification of using Acemedical Acescope

Disposable laryngoscope blades are coated with an anti-fog coating and employ the familiar Macintosh blade technique.

Storage type	Video (MJPEG), Photo (JPG)
Display	2.8*, 240(width) X 320 (height)/TFT-LCD
Alarm	Flashing Pink LED before auto shut down.
Power	Lithium-ion battery (3.6Vdc/1900mAh)
Operating/Charging Time	Over 240 min./over 180 min.
Wireless Charger	:Input 100-240V~, 50/60Hz, 1A
Classification	Class II/Type BF applied part: AceBlade /Internally Powered
Applied part	AceBlade
